# Biomechanical evaluation of a novel integrated artificial axis: A finite element study

**DOI:** 10.1097/MD.0000000000008597

**Published:** 2017-11-27

**Authors:** Yongqiang Zheng, Jianhua Wang, Suixiang Liao, Dongsheng Zhang, Jinshan Zhang, Limin Ma, Hong Xia

**Affiliations:** a Southern Medical University, Guangzhou; b Department of Orthopedics, Jinjiang Municipal Hospital, Jinjiang; c Hospital of Orthopedics, Guangzhou General Hospital of Guangzhou Military Command; d Department of Orthopedics, Panyu Central Hospital, Guangzhou, P. R. China.

**Keywords:** anterior transpedicular screw, atlantoaxial tumor, biomechanics, finite element analysis, prosthesis of axis

## Abstract

Various modified instruments are used for the anterior reconstruction of the tumor lesion affecting the second cervical vertebra, but there have been no reports regarding individual integrated artificial axis (IAA) prosthesis fabricated by selective laser melting. In the present work, a new type of IAA prosthesis has been designed with a 3-dimensional (3D) finite element model of normal occiput-the fourth cervical vertebra being established to assess its biomechanics. For easy comparison, another 3D finite element model is also established for the T-shaped Harms cage and an additional posterior fixation was performed on each model. The models are tested under a preliminary loading of 40 N to simulate cervical physical action including flexion, extension, lateral bending, and rotation. Under various loads from 4 different directions, the maximum stress and displacement of the IAA are less than those of the modified T-shaped Harms cage. Except for flexion, the maximum stress of the third cervical vertebra endplate of the IAA is smaller than that of the modified T-shaped Harms cage. The new prosthesis with axis is a good choice for upper cervical operation, which not only can greatly increase the operation stability of the upper cervical segment but also could significantly reduce the risk of fixation failure due to Harms cage subsidence.

## 1 Introduction

The treatment of tumors affecting the second cervical vertebra (C2) is particularly challenging, because these tumors are rare, with varied types and presentation.^[1]^ The axis is an important part of the craniocervical junction, which transfers the axial load of the 2 lateral masses of the atlas to 3 surfaces on the third cervical vertebra (C3) through the 2 articular facets and the vertebral body.^[2,3]^ Lesions at this level may cause significant morbidity especially when the atlas-axis-junction is destroyed. Under this circumstance, the cervical spinal cord would be progressively compressed, which may cause paralysis, and likely even death.

Surgical treatment of axial tumors always requires total resection and robust reconstruction to re-establish craniocervical stability, and protect the spinal cord at the same time.^[4]^ However, the resection and reconstruction of the axis are among most challenging fields in spine surgery, owing to its craniocervical junction, complex anatomical properties, and the important surrounding nerves and arteries.^[5]^ Currently, resection of the axis can be achieved by the transoral approach, translabiomandibular approach, lateral approach of mastoid process, submandibular carotid triangle approach, and occipitocervical approach.^[6–12]^ With that said, after resection, the reconstruction of the axis is still quite difficult, which is something that orthopedic surgeons and neurosurgeons find rather challenging.

The typical instruments used for reconstruction surgery such as titanium mesh, screws, and plates are not adequate for axis reconstruction, because they are not specifically designed for the unique anatomy of the axis. Even though, aggressive anterior reconstruction techniques have been reported with carefully trimmed titanium mesh cage,^[13]^ C2 prosthesis by integrating the Harms anterior transoral plate and titanium mesh cage,^[2]^ strut graft,^[5]^ titanium mesh cage plus titanium locking plate with the cephalic end of the plate fixed to the first cervical vertebra (C1) anterior arch vertically or unilateral mass obliquely,^[14]^ and modified mesh cage.^[6]^ Although these techniques provide the reconstruction of the ventral defect in the corpus of the axis with the body, fixation failures were reported^[14]^ (e.g., subsidence of the strut graft or fixation breakage). These failures might be because of the difficulty in achieving an adequate connection between the C1 lateral masses located laterally and the supporting C3 vertebral body, thereby resulting in unstable reconstruction.

An individual artificial C2 prosthesis was developed using computer aided design and 3-dimensional (3D) printing based on selective laser melting. The prosthesis could mimic the complex anatomy of the human axis, anatomically matching the neighboring structures to effectively obtain support. In this regard, a novel integrated artificial axis (IAA) combined with anterior transpedicular screw (ATPS) fixation was then successfully developed and its mechanical properties were analyzed using a 3D finite element (FE) method. This new technique is proved to be superior over the T-shaped Harms cage with the anterior overhanging edges fixed to the C1 lateral mass, in terms of both stress distribution and fixation stability, although an additional posterior fixation is unavoidable to improve the stability of anterior reconstruction in both systems.

## 2 Materials and methods

### 2.1 A novel integrated artificial axis prosthesis

**Figure 1:**
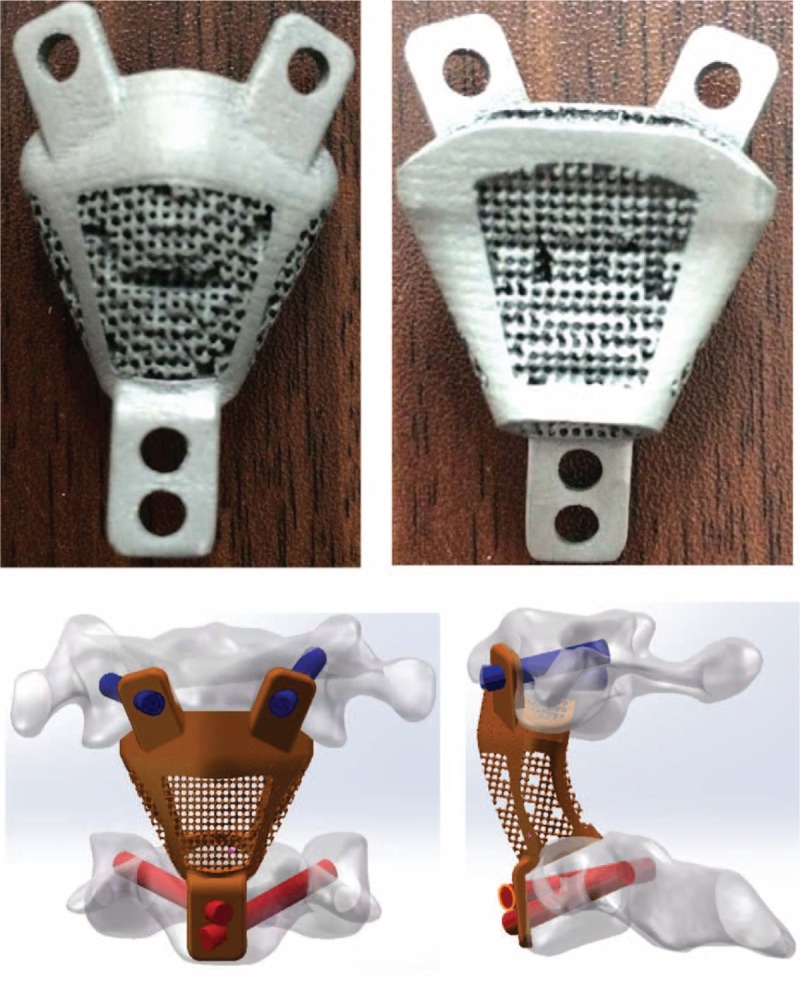
Integrated artificial axis (IAA) fabricated by selective laser melting (SLM) and reconstruction.

The IAA is composed of mass screws of the atlas, ATPSs of the C3, the C2 prosthesis (patent number: CN156100281), and other supporting surgical instruments (Fig. 1).

Preoperatively, 3D anatomical data of the human axis were obtained using the thin slice spinal computed tomography (CT) technique; then, the targeted structure was separated, and the characteristic structure was extracted. The prosthesis was designed to mimic the axial body structures, which simulated the microporous structure of the cancellous bone. The hollow in the center of the prosthesis can be filled with bone graft material to promote the integration, whereas the porous secondary structure on the surface is beneficial for nutrient penetration and bone ingrowth. Next, the vertical integration of the titanium plate fixed branches and screws fixing holes were added. With the metal 3D printing technology, the integrated artificial instrument mimicking the structure of the axis was developed with a Ti-6Al-4V titanium metallic powder. The prosthesis was custom-made for each patient, wherein the size was based on preoperative radiological data measurements.

### 2.2 Generating a finite element model

**Figure 2:**
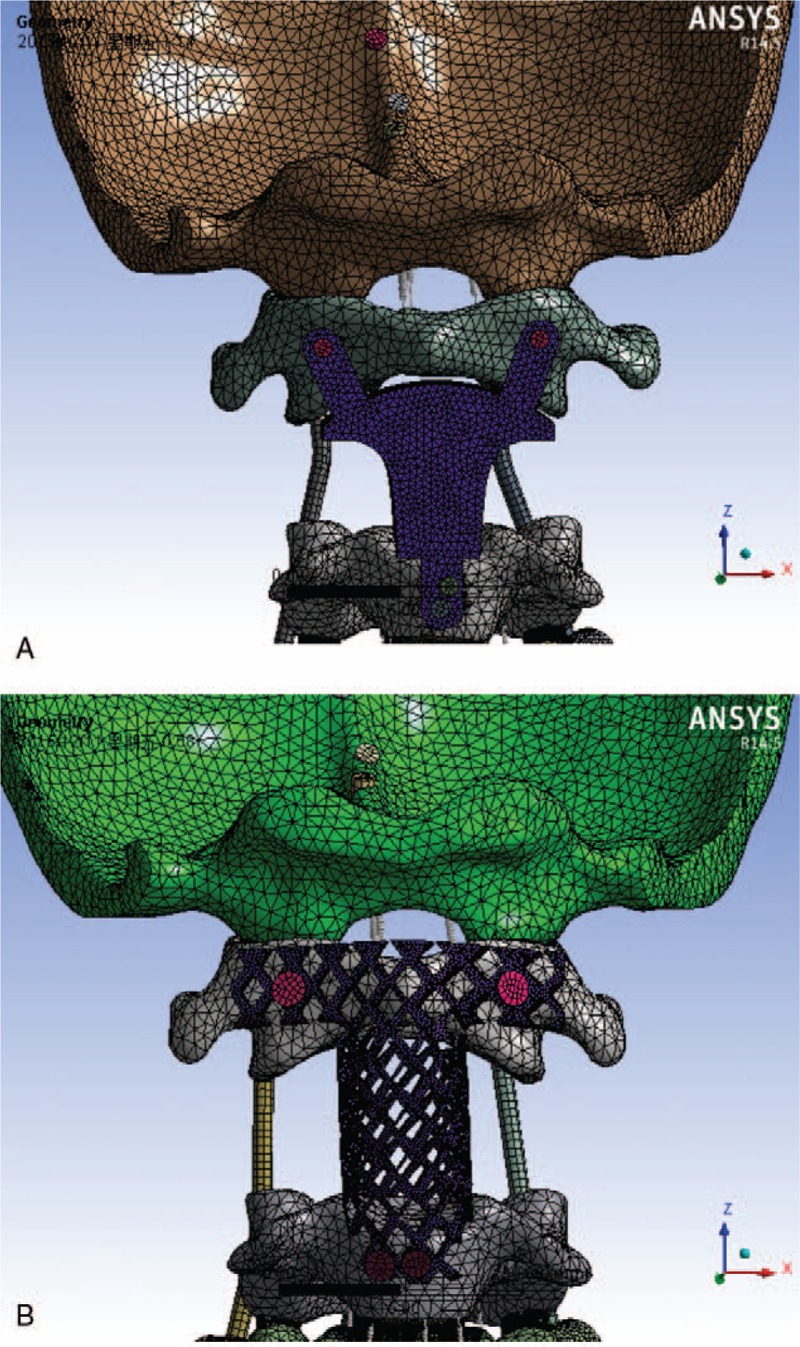
Meshed finite element models of the two systems. A,: The integrated artificial axis (IAA) system. B, The T-shaped Harms cage system.

CT data from the occiput (C0)-the fourth cervical vertebra (C4) region, with a space interval of 0.625 mm (CT provided by Guangzhou General Hospital of Guangzhou Command, Guangzhou, China) were obtained from a 21-year-old healthy male volunteer who was 175 cm tall and weighed 75 kg. The institutional review board (Ethics Committee Guangzhou General Hospital of Guangzhou Military Command. The board's name: Dingcheng Xiang, Yagang Zhao, Bo xie, Cheng Yang, Yuke Chen, Anxing Zhang, Yan Liu, Biao Cheng, Lei Shi, Pingyan Chen, Qingqing Yan, Weiguo Yao) approved the study protocol and the volunteer provided written informed consent. The C0-C4 data were stored in DICOM format. Data were imported into Mimics 10.0 (provided by Biomechanics Laboratory of Southern Medical University) to establish a primary geometric model. The Geomagic Studio 2013 (provided by Biomechanics Laboratory of Southern Medical University, Guangzhou, China) was used to pave and smooth the model. Solidworks 2012 (provided by Biomechanics Laboratory of Southern Medical University, Guangzhou, China) was used to evaluate the model and simulate total axis spondylectomy. Both the models were built after spondylectomy. (Fig. 2, Table 1).

**Table 1:**
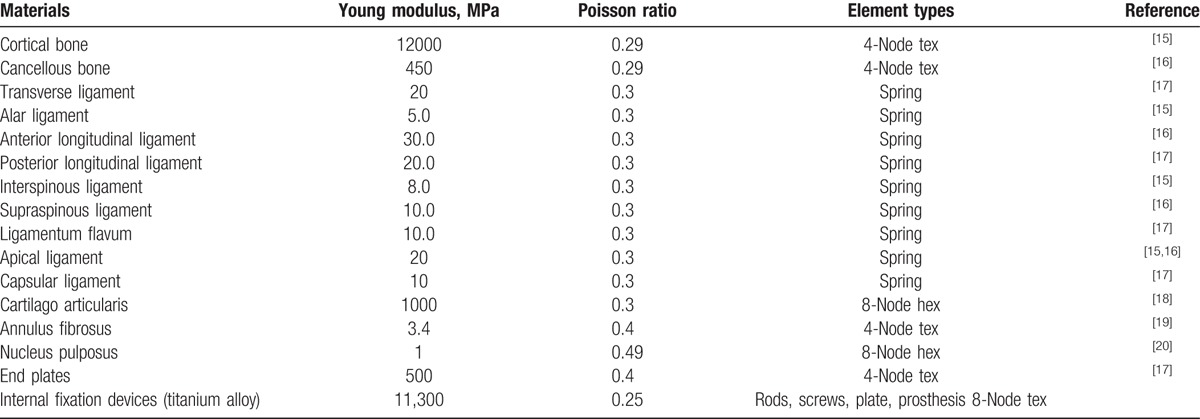
Material properties and element types used for the different tissues of the 2 reconstruction models.

### 2.3 Validation

The model of the IAA consisted of 656,766 nodes and 372,581 units, whereas that of the T-shaped Harms cage system was composed of 672,196 nodes and 377,411 units, using a combined artificial and automatic division method. All ligaments and joint capsules existing in the region were consistent with the vertebral bodies and the vertebral body's cortical bone. Ligaments were simulated as nonlinear, uncompressed, 2-node cable elements, and the corresponding material parameters were assigned to each structure according to the data reported in the literature. The facet articulations of all the joints in our model were simulated as frictional contact elements with a coefficient of 0.1. In addition, an ideal rough behavior was imposed for the surface between the screw and the trajectory. All degrees of freedom were constrained at the base of the C4 vertebra. The range of motion (ROM) was measured and compared with the results published by Panjabi et al.^[21]^ The details of this comparison are shown in Table 2. The data are expressed as the mean ± standard deviation and show that this intact model could be used for further research.

**Table 2:**
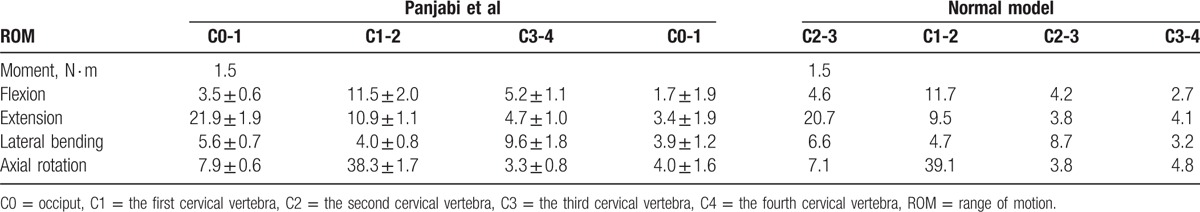
A comparison of the range of motion values (*x* ± *s*).

### 2.4 Boundary and loading condition settings on the models

The same boundary and loading conditions were applied to both models. The superior surface of the occipital condyle was free; however, a boundary condition that constrained all degrees of freedom was applied to the inferior surface of the C4 vertebra. A compressive preload of 40 N^[22]^ combined with a pure moment of 1.5 N · m was applied to the superior surface of the occipital condyle to simulate flexion, extension, lateral bending, and axial rotation. A stress distribution analysis of each fixation technique was performed using ANSYS14.5 (provided by Biomechanics Laboratory of Southern Medical University, Guangzhou, China).

## 3 Results

### 3.1 Stress analysis

**Figure 3:**
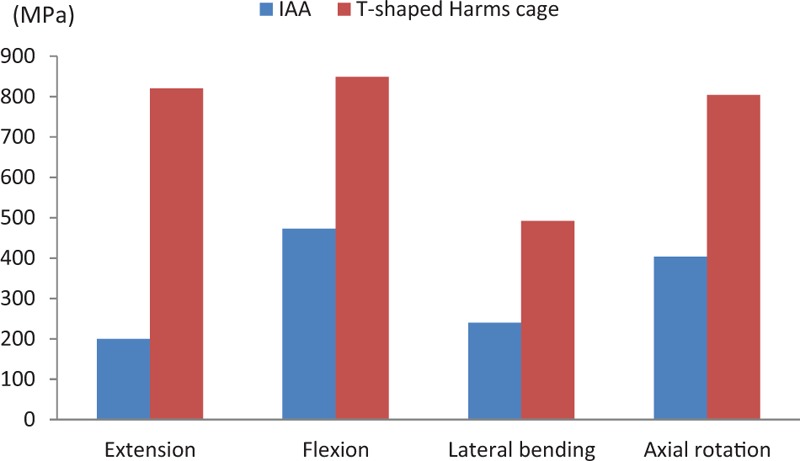
The maximum stress on the 2 fixation devices under loads from 4 different directions. IAA = integrated artificial axis.

**Figure 4:**
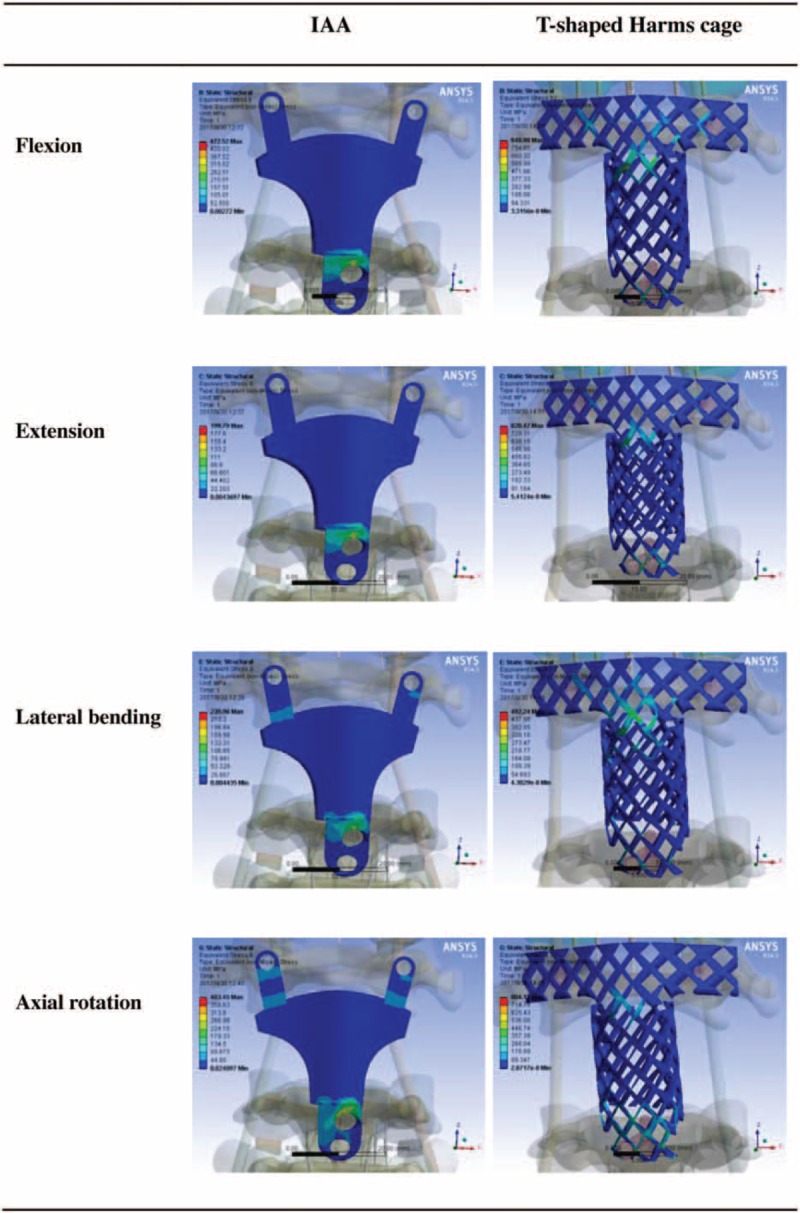
Stress distributions for the integrated artificial axis system and the T-shaped Harms cage system under various loading conditions. IAA = integrated artificial axis.

Various loads were applied to the fixation systems from 4 different directions. The maximum stress on the IAA was determined to be 199.79 MPa in extension, 472.52 MPa in flexion, 239.96 MPa in lateral bending, and 403.45 MPa in axial rotation. By contrast, the maximum stress on the modified T-shaped Harms cage was 820.47 MPa in extension, 848.98 MPa in flexion, 492.24 MPa in lateral bending, and 804.12 MPa in axial rotation. Under various loads from 4 directions, all maximum stress values measured for the IAA system were less than those for the modified T-shaped Harms cage. When compared to those on the modified T-shaped Harms cage, the maximum stress on the IAA system decreased by 75.6%, 44.3%, 51.3%, and 49.8%, respectively, under flexion, extension, lateral bending, and axial rotation (Figs. 3 and 4).

### 3.2 Displacement analysis

**Figure 5:**
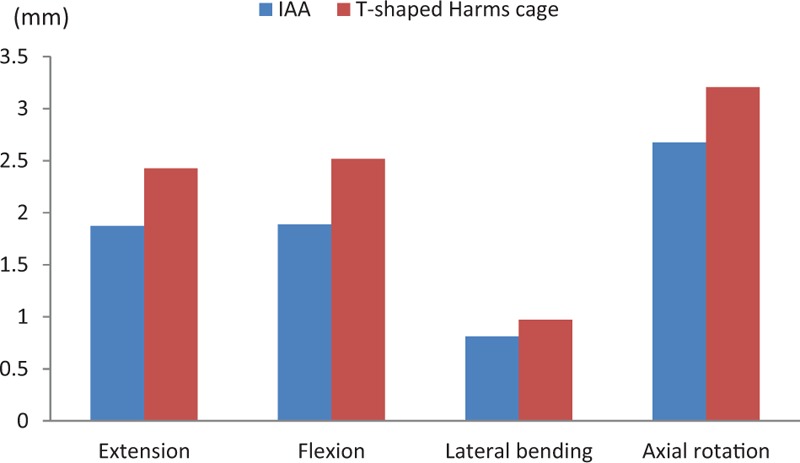
The maximum displacement of the 2 fixation devices under loads from 4 different directions. IAA = integrated artificial axis.

**Figure 6:**
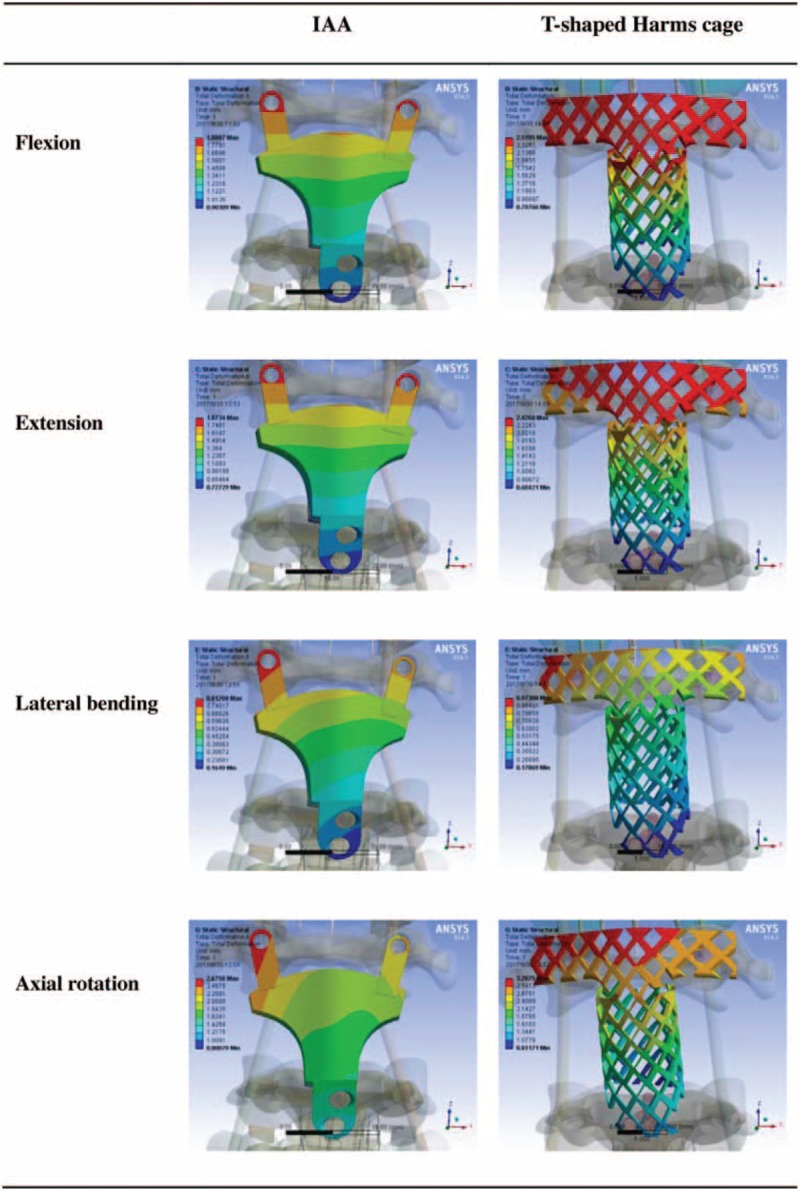
Displacement distributions for the integrated artificial axis system and the T-shaped Harms cage system under various loading conditions. IAA = integrated artificial axis.

Similar to the stress analysis, various loads were applied to both of the fixation systems from 4 different directions. The maximum displacement of the IAA was 1.8734 mm in extension, 1.8887 mm in flexion, 0.8121 mm in lateral bending, and 2.6758 mm in axial rotation. The maximum displacement of the modified T-shaped Harms cage was 2.4268 mm in extension, 2.5195 mm in flexion, 0.9731 mm in lateral bending, and 3.2075 mm in axial rotation. Obviously, the maximum displacements measured for the IAA system were all smaller than those for the modified T-shaped Harms cage. In comparison with those of the modified T-shaped Harms cage, the maximum displacements of the IAA system were reduced by 22.8%, 25.0%, 16.5%, and 16.4%, respectively, under flexion, extension, lateral bending, and axial rotation (Figs. 5 and 6).

### 3.3 Stress of the C3 endplate

**Figure 7:**
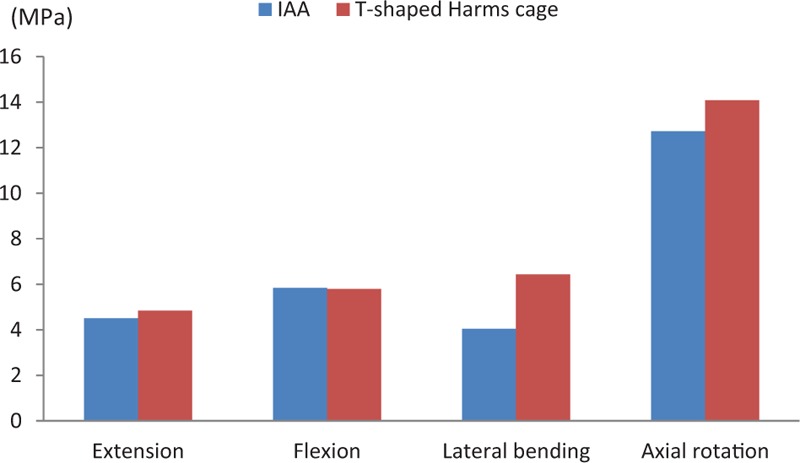
The maximum stress of the third cervical vertebra (C3) endplate for both fixation devices under loads from 4 different directions.

**Figure 8:**
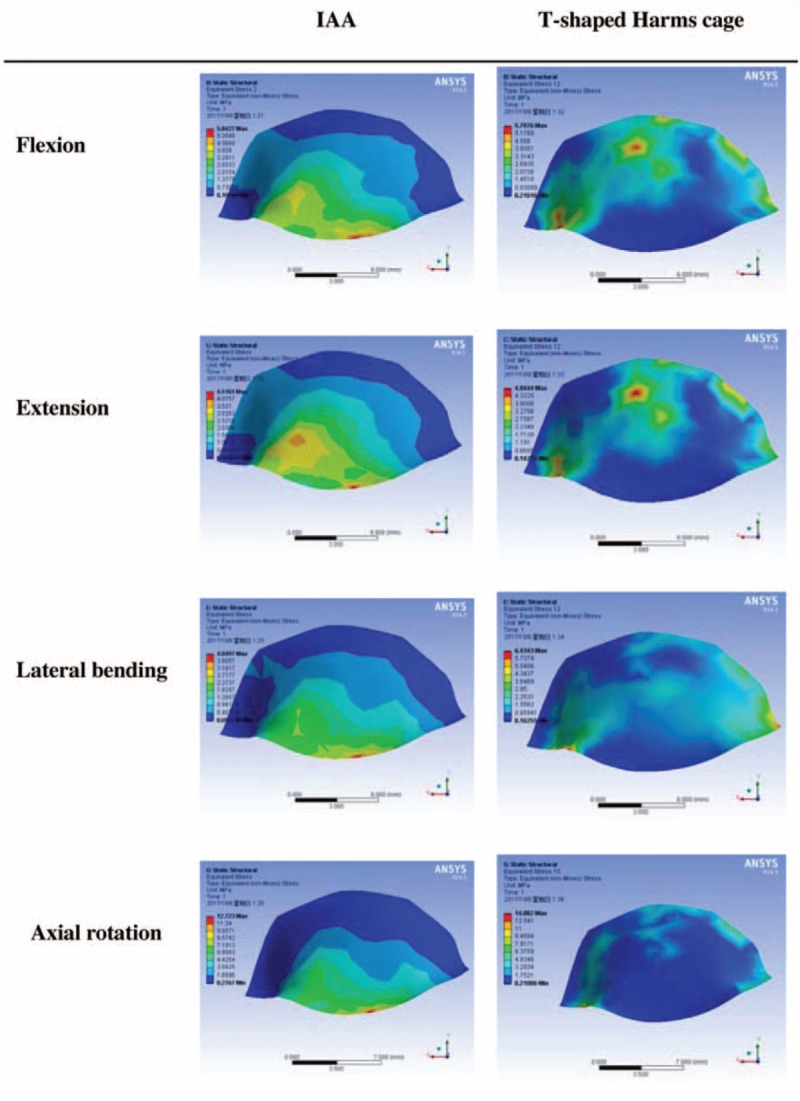
Stress distributions of the third cervical vertebra (C3) endplate for the integrated artificial axis system and the T-shaped Harms cage system under various loading conditions.

The maximum stress of the C3 endplate in the IAA was identified to be 4.5104 MPa in extension, 5.8427 MPa in flexion, 4.0497 MPa in lateral bending, and 12.723 MPa in axial rotation. The maximum stress of the C3 endplate in the modified T-shaped Harms cage was 4.8444 MPa in extension, 5.7976 MPa in flexion, 6.4343 MPa in lateral bending, and 14.082 MPa in axial rotation. In this case, the C3 endplate in the IAA showed 6.9%, 37.1%, and 9.6%, respectively, decrease in the maximum stress under extension, lateral bending, and axial rotation, as compared to the modified T-shaped Harms cage. However, it showed a slight increase (0.8%) of maximum stress under flexion (Figs. 7 and 8).

## 4 Discussion

Surgical treatment of pathological lesions in C2 is a challenge for spine surgeon, because of the anatomical complexity, deep location, and vicinity to vital neurovascular structures in the cranial-cervical junction.

Current treatment strategy for C2 tumor consists of tumor removal, decompression, and reconstruction for defect of C2 structure and instability in the cranial-cervical junction with anterior bone graft, plate, or screws fixation and in combination with posterior instrumentation.

If the vertebral body of axis is defect by the destruction of a pathological process or surgical removal, the instability of cranial-cervical junction may arise. Therefore, posterior fixation is always widely extended to lower cervical so as to compensate for the missing anterior support. Some surgeons also perform anterior reconstructions with bone grafts, titanium cage, screws, and plates. However, the anterior reconstruction alone is basically insufficient, so the posterior occipitocervical fixation and external immobilization are still indispensable.^[23]^

The cranial end of a conventional cage or the bone graft can be secured to the tiny anterior arch or the atlas either by anterior plate or screws. However, the structure is not reliable, because the screws in the anterior arch are too short and not rigid for physical movement. In 2001, Sar et al^[13]^ reported a case of primary osteogenic sarcoma at C2, was treated by anterior transoral resection, fusion, and reconstruction with a modified Harm's cage. In that report, the cage was transformed into a T shape, with a wing in the upside of the cage to secure atlas lateral masses with 2 lateral screws. Of note this modified titanium cage has a better biomechanical strength than the common cylinder harm's cage. In 2007, Jeszenszky et al^[2]^ designed a C2 prosthesis by integrating the Harms anterior transoral plate and titanium mesh cage. Anterior support is provided by the wings of the implant to fix the lateral masses. Three patients underwent treatment for C2 lesions by the custom-made prosthesis, with the follow-up time exceeding 10 years. In 2013, Jandial et al^[24]^ reported a case of posterior approach for axial spondylectomy and circumferential reconstruction with bilateral expandable cages spanning the C1 lateral mass to C3 facet. Biomechanical analysis of different constructs showed that anterior column reconstruction with bilateral cages spanning C1 lateral mass to C3 facet in combination with occipitocervical instrumentation was superior in flexion-extension and equivalent in lateral bending and rotation as compared to currently used constructs. Thus, it is important to support and fix the lateral masses after resection of the axis.

However, the anterior support of modified titanium cage is not reliable either, because the bone graft for the cage with a relative small diameter can only support the thin anterior arch of the atlas, but not the more important lateral mass. Puttlitz et al^[3]^ suggested that the axial load of atlas could be transferred through the C1-C2 facet joints on both sides which are regarded as a 2-column system to axis and subaxial spine. On this basis, the unique role of the body of axis is to redistribute the 2-column axial load of the atlas into a 3-column system of the subaxial spine.

Here, we developed an ideal prosthesis for C2 reconstruction by 3D printing technology, which was custom-made for individual patient according to the presurgery CT scan and measurement. The prosthesis has a bionic shape looks like the body of axis, which can support the lateral mass of atlas by both side of shoulders. The prosthesis is secured with C1 by 2 lateral screws and the bottom of prosthesis is connected with end plate of C3 by 2 screws.

The ATPS fixation was first introduced by Aramomi et al^[25]^ and successfully applied in 9 cases. To date, clinical feasibility of ATPS fixation has been reported several times.^[26–29]^ The entry point for C3 is in the opposite side of the pedicle with screw, 2 mm from the median sagittal plane, 7 to 8 mm from the upper end plate, with the extraversion angle of 47° to 48° and the declination angle of 8° to 23°. The pedicle screw diameter of C3 is 3.5 mm, with the screw length of 30 mm. By this technique, the plate or prosthesis could be fixed more tightly than common vertebra screws. In our prosthesis, the anterior reverse screw can be used for fixation with C3. In order to direct the trajectory of the C3 screws, the computer-assisted design 3D printing pedicle screw guide could be used in the procedures of prosthesis implantation.

In this research, we successfully established 2 FE reconstruction models, that is, modified T-shaped titanium cage and 3D printing C2 prosthesis, combined with same posterior fixation. Under the application of various loads from 4 different directions, we found that the maximum stress of the IAA system was lower than that of the modified Harm's titanium cage, which indicates that the stress of the IAA is more reasonably dispersed. The maximum stress of the modified T-shaped harms cage was approaching the yield strength of titanium alloy, which implies that failure is likely to happen if the axis is reconstructed with the modified titanium cage.

Similarly, it is found that the maximum displacement with the IAA system was less than that with the modified titanium cage, which suggests that reconstruction of the axis with the IAA combined with the posterior fixation has a better stability than modified harm's titanium cage, thereby providing a better mechanical environment for the graft fusion.

Subsidence is inherent in the interbody fusion process and is defined as the sinking of a body with a higher elasticity modulus (e.g., graft, cage, spacer) into a body characterized by a lower elasticity modulus (e.g., vertebral body), resulting in changes in spinal geometry. Because cage subsidence most commonly occurs at the upper endplate of the lower vertebra at the operated segment, only the maximum stress of the C3 endplate was calculated.^[30]^ We found that the maximum stress of the C3 endplate on both systems was less than the yield strength of titanium alloy (104–208 MPa), indicating that subsidence is unlikely to occur. Except for an increase of 0.8% under flexion, we found that the maximum stress of the C3 endplate for the IAA system was less than that for the modified T-shaped Harms cage.

### 4.1 Study limitations

There are still some potential limitations to this study. First, the model of resecting the axis tumor was assumed by removing the axis and all the relative ligament elements, which did not closely approximate the actual condition. It is a special challenge to model various conditions of axis tumor. Second, this study used the acquisition of skull base and neck CT data of a single healthy male adult volunteer to design the artificial axis prosthesis, which could not be widely applied to other patients owing to the different shape, length, width, height, and orientation. Changes in size of the prosthesis may lead to different results in terms of ROM and stress distribution. Third, several simplifications had to be made, because the model includes a huge region and complicated reconstruction implants. Finally, for both reconstructions, the screws were tightly locked to the bone, implants and bony endplate to ensure perfect surface-to-surface contact in the FE study. This assumption would result in a smaller ROM than that in a clinical trial. Thus, additional cadaveric studies are required in the future to further validate this model.^[31]^

## 5 Conclusion

The IAA system not only can greatly increase the operation stability of the upper cervical segment but also could significantly reduce the risk of fixation failure due to Harms cage subsidence.

## References

[1] Jeszenszky DJ Haschtmann D Probstl O Tumors and metastases of the upper cervical spine (C0-2). A special challenge [in German]. Orthopade 2013;42:746–54. 23989592 10.1007/s00132-013-2069-1

[2] Jeszenszky D Fekete TF Melcher R C2 prosthesis: anterior upper cervical fixation device to reconstruct the second cervical vertebra. Eur Spine J 2007;16:1695–700. 17632736 10.1007/s00586-007-0435-6 PMC2078292

[3] Puttlitz CM Harms J Xu Z A biomechanical analysis of C2 corpectomy constructs. Spine J 2007;7:210–5. 17321971 10.1016/j.spinee.2006.05.016

[4] Ortega-Porcayo LA Cabrera-Aldana EE Arriada-Mendicoa N Operative technique for en bloc resection of upper cervical chordomas: extended transoral transmandibular approach and multilevel reconstruction. Asian Spine J 2014;8:820–6. 25558326 10.4184/asj.2014.8.6.820 PMC4278989

[5] Matsumoto M Watanabe K Ishii K Complicated surgical resection of malignant tumors in the upper cervical spine after failed ion-beam radiation therapy. Spine (Phila Pa 1976) 2010;35:E505–9. 20421861 10.1097/BRS.0b013e3181caa86c

[6] Wu W Li F Fang Z Total spondylectomy of C2 and circumferential reconstruction via combined anterior and posterior approach to cervical spine for axis tumor surgery. J Huazhong Univ Sci Technolog Med Sci 2013;33:126–32. 23392721 10.1007/s11596-013-1084-0

[7] Suchomel P Barsa P Single stage total endolesional C2 spondylectomy for chordoma. Eur Spine J 2013;22:1453–6. 23901402 10.1007/s00586-013-2813-6 PMC3676555

[8] Stulik J Kozak J Sebesta P Total spondylectomy of C2: a new surgical technique. Acta Chir Orthop Traumatol Cech 2007;74:79–90. 17493408

[9] Stulik J Kozak J Sebesta P Total spondylectomy of C2: report of three cases and review of the literature. J Spinal Disord Tech 2010;23:e53–8. 21131798 10.1097/BSD.0b013e3181d0c1e5

[10] Rhines LD Fourney DR Siadati A En bloc resection of multilevel cervical chordoma with C-2 involvement. Case report and description of operative technique. J Neurosurg Spine 2005;2:199–205. 15739534 10.3171/spi.2005.2.2.0199

[11] Eleraky M Setzer M Vrionis FD Posterior transpedicular corpectomy for malignant cervical spine tumors. Eur Spine J 2010;19:257–62. 19823877 10.1007/s00586-009-1185-4 PMC2899823

[12] Suchomel P Buchvald P Barsa P Single-stage total C-2 intralesional spondylectomy for chordoma with three-column reconstruction. Technical note. J Neurosurg Spine 2007;6:611–8. 17561755 10.3171/spi.2007.6.6.17

[13] Sar C Eralp L Transoral resection and reconstruction for primary osteogenic sarcoma of the second cervical vertebra. Spine (Phila Pa 1976) 2001;26:1936–41. 11568710 10.1097/00007632-200109010-00025

[14] Yang X Wu Z Xiao J Sequentially staged resection and 2-column reconstruction for C2 tumors through a combined anterior retropharyngeal-posterior approach: surgical technique and results in 11 patients. Neurosurgery 2011;69:ons184–93. 21499150 10.1227/NEU.0b013e31821bc7f9

[15] Panjabi MM Oxland TR Parks EH Quantitative anatomy of cervical spine ligaments. Part I. Upper cervical spine. J Spinal Disord 1991;4:270–6. 1802157

[16] Panjabi MM Oxland TR Parks EH Quantitative anatomy of cervical spine ligaments. Part II. Middle and lower cervical spine. J Spinal Disord 1991;4:277–85. 1802158 10.1097/00002517-199109000-00004

[17] Yoganandan N Kumaresan S Pintar FA Biomechanics of the cervical spine Part 2. Cervical spine soft tissue responses and biomechanical modeling. Clin Biomech (Bristol, Avon) 2001;16:1–27. 10.1016/s0268-0033(00)00074-7 11114440

[18] Womack W Woldtvedt D Puttlitz CM Lower cervical spine facet cartilage thickness mapping. Osteoarthritis Cartilage 2008;16:1018–23. 18308589 10.1016/j.joca.2008.01.007

[19] Zhang QH Teo EC Ng HW Finite element analysis of moment-rotation relationships for human cervical spine. J Biomech 2006;39:189–93. 16271604 10.1016/j.jbiomech.2004.10.029

[20] Yoganandan N Kumaresan SC Voo L Finite element modeling of the C4-C6 cervical spine unit. Med Eng Phys 1996;18:569–74. 8892241 10.1016/1350-4533(96)00013-6

[21] Panjabi M Dvorak J Duranceau J Three-dimensional movements of the upper cervical spine. Spine (Phila Pa 1976) 1988;13:726–30. 3194778 10.1097/00007632-198807000-00003

[22] Folsom MD Hodgson E Biochemical characteristics of insect microsomes: NADPH oxidation by intact microsomes from the housefly, Musca domestica. Comp Biochem Physiol 1970;37:301–10. 4395653 10.1016/0010-406x(70)90558-x

[23] Shin H Barrenechea IJ Lesser J Occipitocervical fusion after resection of craniovertebral junction tumors. J Neurosurg Spine 2006;4:137–44. 16506481 10.3171/spi.2006.4.2.137

[24] Jandial R Kelly B Bucklen B Axial spondylectomy and circumferential reconstruction via a posterior approach. Neurosurgery 2013;72:300–8. 23149951 10.1227/NEU.0b013e31827b9d38 PMC3883459

[25] Aramomi M Masaki Y Koshizuka S Anterior pedicle screw fixation for multilevel cervical corpectomy and spinal fusion. Acta Neurochir (Wien) 150:575–82. 18431528 10.1007/s00701-008-1574-1

[26] Koller H Hempfing A Acosta F Cervical anterior transpedicular screw fixation. Part I: Study on morphological feasibility, indications, and technical prerequisites. Eur Spine J 2008;17:523–38. 18224358 10.1007/s00586-007-0572-y PMC2295270

[27] Koller H Acosta F Tauber M Cervical anterior transpedicular screw fixation (ATPS)—part II. Accuracy of manual insertion and pull-out strength of ATPS. Eur Spine J 2008;17:539–55. 18224357 10.1007/s00586-007-0573-x PMC2295271

[28] Koller H Hitzl W Acosta FT In vitro study of accuracy of cervical pedicle screw insertion using an electronic conductivity device (ATPS part III). Eur Spine J 2009;18:1300–13. 19575244 10.1007/s00586-009-1054-1 PMC2899545

[29] Zhao L Li G Liu J Radiological studies on the best entry point and trajectory of anterior cervical pedicle screw in the lower cervical spine. Eur Spine J 2014;23:2175–81. 25056398 10.1007/s00586-014-3473-x

[30] Zhang BC Liu HB Cai XH Biomechanical comparison of a novel transoral atlantoaxial anchored cage with established fixation technique - a finite element analysis. BMC Musculoskelet Disord 2015;16:261. 26395763 10.1186/s12891-015-0662-7 PMC4579577

[31] Puttlitz CM Goel VK Traynelis VC A finite element investigation of upper cervical instrumentation. Spine (Phila Pa 1976) 2001;26:2449–55. 11707709 10.1097/00007632-200111150-00011

